# Association between Polypharmacy and Adverse Events in Patients with Alzheimer’s Disease: An Analysis of the Japanese Adverse Drug Event Report Database (JADER)

**DOI:** 10.3390/medicina60101633

**Published:** 2024-10-06

**Authors:** Nobuhiro Otani, Kanae Kanda, Nlandu Roger Ngatu, Akitsu Murakami, Yusuke Yamadori, Tomohiro Hirao

**Affiliations:** 1 Department of Public Health, Faculty of Medicine, Kagawa University, Miki 761-0793, Kagawa, Japan; kanda.kanae@kagawa-u.ac.jp (K.K.); ngatu.nlandu@kagawa-u.ac.jp (N.R.N.); 2 Cancer Center, Kagawa University, Miki 761-0793, Kagawa, Japan; murakami.akitsu@kagawa-u.ac.jp; 3 Department of Anesthesiology, Faculty of Medicine, Kagawa University, Miki 761-0793, Kagawa, Japan; yamadori.yusuke@kagawa-u.ac.jp

**Keywords:** Alzheimer’s disease, polypharmacy, acetylcholinesterase inhibitors: NMDA receptor antagonists, Japanese Adverse Drug Event Report (JADER)

## Abstract

*Background and Objectives*: Alzheimer’s disease is a global health concern, with a rising prevalence among the elderly. Current pharmacological treatments, including acetylcholinesterase inhibitors (AChEIs) and N-Methyl D-Aspartate (NMDA) receptor antagonists, are associated with adverse events (AEs), particularly in the context of polypharmacy. This study aimed to investigate the relationship between Alzheimer’s disease treatment combinations, the number of concomitant medications, and the occurrence of AEs. *Materials and Methods:* Data from the Japanese Adverse Drug Event Report database, spanning from April 2004 to June 2020, were analyzed. Patients aged 60 and older with Alzheimer’s disease treated with AChEIs (donepezil, galantamine, and rivastigmine) or the NMDA receptor antagonist memantine were included. Logistic regression models were employed to assess the association between AEs and Alzheimer’s disease drug combinations, as well as the number of concomitant medications. *Results:* Among 2653 patients, 47.7% were prescribed five or more drugs. The frequency of AEs was 6.4% for bradycardia, 4.6% for pneumonia, 3.6% for altered state of consciousness, 3.5% for seizures, 3.5% for decreased appetite, 3.5% for vomiting, 3.4% for loss of consciousness, 3.4% for fracture, 3.2% for cardiac failure, and 3.0% for falls. The combination of memantine with AChEIs was associated with a higher risk of bradycardia, whereas donepezil alone was linked to a reduced risk of fractures and falls. Polypharmacy was significantly correlated with an increased incidence of AEs, particularly altered state of consciousness, decreased appetite, vomiting, and falls. The adjusted odds ratios for using five or more drugs compared to no drugs was 10.45 for altered state of consciousness, 7.92 for decreased appetite, 4.74 for vomiting, and 5.95 for falls. *Conclusions:* In the treatment of Alzheimer’s disease, the occurrence of AEs is associated with the number of concurrent medications, independently of the known AEs of Alzheimer’s disease drugs and their combination patterns.

## 1. Introduction

Dementia is a leading cause of cognitive impairment in the elderly, with an estimated global prevalence exceeding 55 million individuals [1]. There are many conditions that cause dementia (Table 1); Alzheimer’s disease accounts for 60–70% of all dementia cases [1,2,3]. Alzheimer’s disease is characterized by two pathological changes, amyloid deposition and neurofibrillary tangles, which cause neuronal death, synapse depletion, and decreased acetylcholine in the cerebral cortex, hippocampus, and frontobasal region, leading to the development of dementia [4]. The number of patients is projected to double every 20 years, reaching 74.7 million by 2030 and 131.5 million by 2050 [5]. In Japan, the number of elderly individuals with dementia aged 65 and above was estimated to be 4.62 million in 2012 [6]. This number is projected to rise to between 6.75 and 7 million by 2025, and to between 8 and 9.5 million by 2040 [7].

Efforts to develop treatments for dementia, a condition with a significant social burden, have been ongoing. In 2022, a new drug, lecanemab, was introduced. However, its indication is limited to early Alzheimer’s disease, leaving many patients with dementia without a targeted therapy [8]. The current standard pharmacological treatment consists of four drugs: three acetylcholinesterase inhibitors (AChEIs)—donepezil, galantamine, and rivastigmine—and the N-Methyl D-Aspartate (NMDA) receptor antagonist memantine [4]. AChEIs inhibit the enzymatic hydrolysis of acetylcholine and maintain cholinergic neuronal signaling. NMDA receptor antagonists modulate glutamate signaling and block the uptake of excess calcium, which is thought to cause neuronal deterioration. They are known to be effective in temporarily alleviating symptoms, but do not have a fundamental therapeutic effect [4] (Table 2).

As most patients with Alzheimer’s disease are elderly, they often present with comorbidities [9,10]. This leads to significant concerns about polypharmacy, especially given the increased risk of polypharmacy in patients with Alzheimer’s disease [11,12]. Polypharmacy is the concurrent use of multiple medications; while there is no standard definition, it is often defined as the routine use of five or more medications [13,14]. The mechanisms of polypharmacy include pharmacokinetic and pharmacodynamic interactions. In pharmacokinetic interactions, the absorption, distribution, metabolism, and excretion of a drug are affected by other drugs, resulting in changes in blood concentrations that cause excessive effects (intoxication) or weakening of effects. Pharmacodynamic interactions are caused by interactions at the site of action, such as receptors, or by the overlap of drug effects, resulting in enhanced or weakened effects. As the number of concomitant medications increases, so does the risk of polypharmacy [15]. Additionally, commonly used medications for Alzheimer’s disease, such as AChEIs and NMDA receptor antagonists, are associated with adverse events, including syncope, bradycardia, and nausea/vomiting [16,17,18]. The risk of these adverse events varies depending on the specific combination of the four drugs used [19,20,21].

The impact of Alzheimer’s disease treatment combinations and the number of concomitant medications on the risk of adverse events remains unclear. Therefore, this study aimed to investigate the effect of various combinations of the four major Alzheimer’s disease drugs and the number of concurrent medications on the incidence of adverse events, using data derived from JADER, a publicly available adverse event database. JADER is an adverse drug event database for Japan, which has a large elderly population, and is suitable for the analysis of age-related diseases such as Alzheimer’s disease.

## 2. Materials and Methods

### 2.1. Data Source

This study employed a large database for exploratory analysis. We analyzed data from the Japanese Adverse Drug Event Report (JADER) database spanning April 2004 and June 2020. JADER is the spontaneous adverse drug event reporting system of Japan, collecting data submitted by pharmaceutical companies and medical professionals to the Ministry of Health, Labour, and Welfare. The information, with personal identifiers removed, is publicly accessible through the Pharmaceuticals and Medical Devices Agency website (http://www.pmda.go.jp accessed on 8 August 2024). JADER serves as the largest drug adverse event database in Japan and has been extensively utilized for research on drug-related adverse events [19,22,23,24].

The JADER database consists of four linked tables: (1) a table on demographics, including age and sex; (2) a table on drug information, which classifies medications into three categories based on their involvement in adverse events (suspected drugs, concomitant drugs, and interacting drugs); (3) a table on adverse event information; and (4) a table on underlying diseases. Each table is linked by a unique case identification number, ensuring anonymity.

### 2.2. Data Processing

The study population included patients aged 60 years or older diagnosed with Alzheimer’s disease. The drugs of interest were AChEIs (donepezil, galantamine, and rivastigmine) and the NMDA receptor antagonist memantine. In Japan, rivastigmine was approved exclusively as a transdermal patch in 2010, with no prior use in any form before this approval. Cases involving these drugs, either as suspected or interacting drugs, were included.

**Figure 1:**
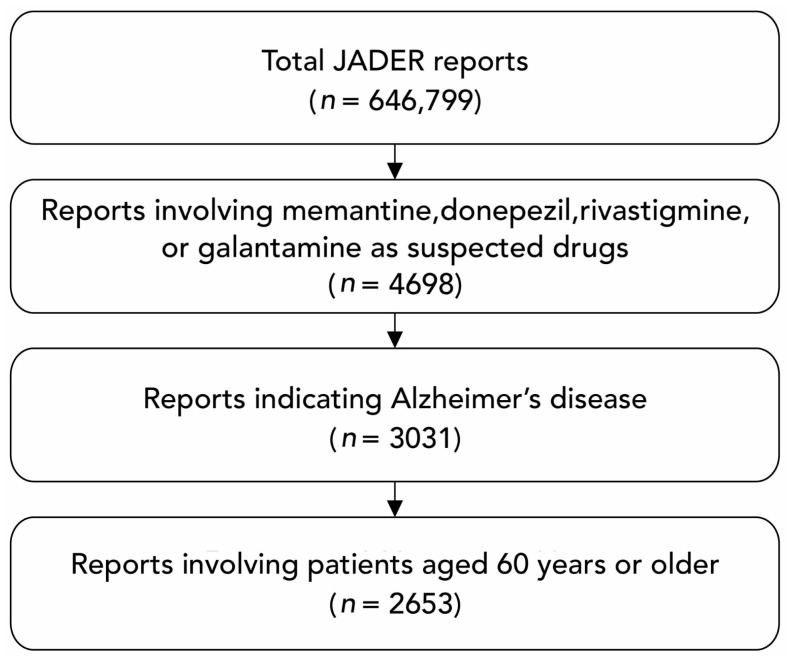
Flowchart of the dataset construction for the analysis. JADER, Japanese Adverse Drug Event Report.

From the total of 646,779 case reports registered in the JADER database, 4698 reports involving donepezil, rivastigmine, galantamine, or memantine were identified. Of these, 2653 cases of Alzheimer’s disease in patients aged 60 years or older were included, whereas those with incomplete data were excluded (Figure 1).

### 2.3. Adverse Events

Adverse events were classified according to the Preferred Terms (PTs) in the Japanese version of the Medical Dictionary for Regulatory Activities/Japanese version 23.1. The ten most frequently reported adverse events associated with Alzheimer’s disease treatment were selected for analysis.

### 2.4. Variables

The primary evaluation variables were “combination with Alzheimer’s disease therapeutics” and “the number of concomitant medications used.” The combination of Alzheimer’s disease therapeutic agents was categorized into five patterns: monotherapy with AChEIs (donepezil, rivastigmine, and galantamine) or the NMDA receptor antagonist memantine, and combination therapy (memantine + AChEI). Additionally, the number of concomitant medications used, excluding Alzheimer’s disease treatments, was classified into six categories based on the number of drugs used: zero drugs, one drug, two drugs, three drugs, four drugs, and five drugs or more.

Due to the limitations of the JADER database, the number of variables that can be used is limited, so in this study, sex, age, and comorbidities were used as covariates to adjust for confounding factors. Age was classified into four categories: 60–69 years, 70–79 years, 80–89 years, and 90 years and older. Comorbidities were selected from the most prevalent conditions among the 2653 cases, including hypertension, diabetes mellitus, hyperlipidemia, malignant neoplasms, and cardiovascular diseases such as ischemic heart disease and cerebrovascular disease, as well as neuropsychiatric disorders such as depression, Parkinson’s disease, and sleep disorders. These include risk factors for Alzheimer’s disease.

### 2.5. Statistical Analysis

This exploratory analysis utilized a large real-world database, and sample size estimation was not conducted. Statistical analyses were performed using logistic regression models, both bivariate and multivariate, with adverse events as the outcome variable. In the multivariate model, adjustments were made for sex, age, and the presence of comorbidities, in addition to the primary variables of interest: combination with Alzheimer’s disease therapeutics and the number of concomitant medications used. For the combination of Alzheimer’s disease therapeutics, memantine alone served as the reference drug for comparison with other therapeutic patterns. Odds ratios (ORs) were used as measures of association, and statistical significance was determined using the Wald test with a 5% significance level. The analyses were conducted using JMP Pro 17.0 (SAS Institute Inc., Cary, NC, USA).

### 2.6. Ethical Considerations

This study utilized anonymized open data which did not contain any personally identifiable information. Consequently, no ethical concerns were associated with the research. All analytical procedures adhered to the principles of the Declaration of Helsinki and the manuscript preparation followed STROBE guidelines.

## 3. Results

### 3.1. Characteristics of the Study Population

Among the 2653 participants analyzed, 60.2% were women. The most common age group was that of 80–89-year-old patients, comprising 53.5% of the sample. Regarding Alzheimer’s disease treatments, donepezil was the most frequently prescribed at 41%, followed by rivastigmine (25%), galantamine (17.3%), combination therapy (memantine + AChEI) (8.9%), and memantine alone (7.8%). In terms of the number of concomitant medications, 17.5% of participants did not take additional drugs, while 10.2%, 8.6%, 10.0%, 6.1%, and 47.7% took one, two, three, four, and five or more additional medications, respectively (Table 3).

Comorbid conditions included hypertension (35.2%), hyperlipidemia (13.6%), diabetes mellitus (12.3%), cerebrovascular disease (10.7%), sleep disorders (7.4%), ischemic heart disease (6.1%), depression (4.7%), Parkinson’s disease (4.3%), and cancer (3.1%) (Table 3). The most frequently reported adverse events were bradycardia (PT code: 10006093; 6.4%), pneumonia (PT code: 10035664; 4.6%), altered state of consciousness (PT code: 10001854; 3.6%), seizures (PT code: 10039906; 3.5%), decreased appetite (PT code: 10061428; 3.5%), vomiting (PT code: 10047700; 3.5%), loss of consciousness (PT code: 10024855; 3.4%), fractures (PT code: 10017070; 3.4%), cardiac failure (PT code: 10007554; 3.2%), and falls (PT code: 10016173; 3.0%) (Table 4).

### 3.2. Association between Alzheimer’s Disease Treatment Combinations and Adverse Events

The treatment patterns for Alzheimer’s disease demonstrated a significant association with adverse events, including bradycardia, pneumonia, vomiting, loss of consciousness, fractures, and falls. These results were statistically significant in both bivariate and multivariate analyses, indicating that the associations were independent of sex, age, the presence of comorbidities, and the number of other medications used.

Compared to memantine monotherapy, the adjusted ORs (AORs) for bradycardia were 3.07 for donepezil, 2.00 for rivastigmine, 3.12 for galantamine, and 2.49 for the combination of memantine and an AChEI, suggesting an increased risk of occurrence. For pneumonia, the AORs were 0.37 for donepezil and 0.19 for the combination of memantine and an AChEI compared to memantine monotherapy. Regarding vomiting, the AORs were higher with rivastigmine and galantamine compared to memantine monotherapy, at 3.56 and 2.10, respectively, whereas the AOR was only 0.63 when memantine was combined with an AChEI. For loss of consciousness, the AORs of donepezil and rivastigmine were lower compared to memantine alone, at 0.15 and 0.34, respectively. For both fractures and falls, the AOR for donepezil was 0.28, which was lower than that for memantine monotherapy, indicating a reduced risk of occurrence (Table 5).

### 3.3. Association between the Number of Concomitant Medications and Adverse Events

The relationship between the number of concomitant drugs and the occurrence of adverse events was significant for an altered state of consciousness, decreased appetite, vomiting, and falls. These results were statistically significant in both bivariate and multivariate analyses, indicating that the associations were independent of sex, age, the presence of comorbidities, and combination Alzheimer’s disease therapy.

The AORs for altered state of consciousness, compared to no concomitant drug use, were 6.93, 5.90, 17.53, 6.80, and 10.45 for one, two, three, four, and five or more drugs, respectively. For decreased appetite, the AORs were 5.06, 4.31, 3.66, 9.72, and 7.92 for one, two, three, four, and five or more drugs, respectively. For vomiting, the AORs were 3.11, 7.87, 3.78, 6.85, and 4.74 for one, two, three, four, and five or more drugs, respectively. For falls, the AORs were 5.33, 3.04, 4.49, 1.72, and 5.95 for one, two, three, four, and five or more drugs, respectively. The risk of adverse events was higher compared to when no concomitant drugs were used (Table 5).

## 4. Discussion

Combined Alzheimer’s disease therapy and the number of concomitant medications used are associated with adverse events [11,12,19,20,21]. In this study, we observed that these factors independently serve as risk factors.

The association between combined Alzheimer’s disease therapy and various adverse events largely aligns with previous studies. Bradycardia and vomiting, known side effects of AChEIs, were similarly noted in this study [16,17,18,19,20,21]. The AOR of bradycardia was 2.49 for the combination of memantine and AChEIs, indicating a higher risk compared to memantine alone. Due to the characteristics of the database, it is not possible to apply the AOR to actual clinical practice, but it is thought to indicate the existence and trends of risk. With regard to vomiting, there was no increase in risk with this combination, and the results were consistent with a study by Shi et al. [21].

In cases of loss of consciousness, the AORs of donepezil and rivastigmine were lower compared to memantine alone. Furthermore, the AORs of fractures and falls were lower with donepezil. These suggest a higher incidence of these adverse events with memantine, which is consistent with the existing knowledge [16,17,18]. A systematic review of adverse events associated with AChEIs found no significant association between donepezil and overall adverse events [25], suggesting that donepezil use may be relatively safe concerning these adverse events. However, to our knowledge, studies examining the association of these adverse events with Alzheimer’s disease treatments are lacking, highlighting the need for further research.

For pneumonia, the combination therapy of memantine with an AChEI, as well as donepezil, was associated with a lower risk compared to memantine alone. On the other hand, rivastigmine was associated with higher risk compared to memantine alone. Lampela et al. [26] reported in a Finnish nationwide cohort study that rivastigmine patch and memantine have a higher risk of pneumonia than donepezil, which is consistent with the results of this study. While our study adjusted for age and comorbidities, other contributing factors may exist, and further research is needed to elucidate these underlying mechanisms.

The number of concomitant medications was associated with adverse events, such as altered state of consciousness, decreased appetite, vomiting, and falls. While no universally accepted definition is available for polypharmacy, there are reports that the incidence of adverse drug reactions increases with the use of six or more drugs [27], and that people taking five or more drugs are more likely to suffer a fall than those taking four or fewer [28]. Furthermore, since it has been reported that the risk of polypharmacy increases linearly with the number of concomitant medications [15], we adopted the definition of the World Health Organization: concurrent use of multiple medications and commonly as the routine use of five or more drugs [13].

Among the 10 adverse events analyzed, bradycardia, altered state of consciousness, decreased appetite, vomiting, loss of consciousness, and falls are known side effects of Alzheimer’s disease drugs. Of these, both the combination with Alzheimer’s disease therapeutics and the number of concomitant medications used were statistically significant for vomiting and falls, whereas only the combination was significant for bradycardia and loss of consciousness, and only the number of drugs was significant for altered state of consciousness and decreased appetite. In the case of bradycardia and loss of consciousness, it is possible that the effects of Alzheimer’s disease drugs are more pronounced, while the effects of polypharmacy are less pronounced. A similar trend may be inferred for altered consciousness and decreased appetite. To fully elucidate these findings, it is imperative to evaluate not only the number of concomitant medications but also the specific agents involved, which remains a critical area for future research.

In this study, we demonstrated a significant correlation between the number of concurrent medications, irrespective of their types, and the occurrence of adverse events. Numerous studies have addressed polypharmacy and comprehensive lists of primary symptoms and frequently implicated drugs have been compiled [29]. In the elderly, well-documented evidence linking central nervous system-related symptoms to an increased risk of falls and fractures is available [30,31,32,33,34].

This study revealed that in Alzheimer’s disease treatment, an association exists between the number of concomitant medications and adverse events, independent of the known side effects of Alzheimer’s disease drugs. While it is generally accepted that an increased number of medications correlates with a higher incidence of adverse events [13,29], our study observed this trend; however, it did not identify a clear dose–response relationship.

This study has several limitations. First, the analyzed database relies on voluntary reporting, which does not encompass all adverse events; both over- and under-reporting are therefore possible. Second, as this study utilized a database of adverse events, cases without adverse events were not included. As a result, this study does not cover the entire population of Alzheimer’s disease patients who received drug treatment, and the magnitude of the AOR and other indicators cannot be immediately applied to real-world clinical practice. However, the observed trends are likely to be consistent, and they may be applicable in comparisons with reference drugs. Third, due to database constraints, we were unable to adjust for confounding factors beyond age and comorbidities. For instance, the analysis did not account for psychotropic drugs that could be causative agents. In addition, there is no information on the patient’s condition, such as the stage of Alzheimer’s disease or performance status.

It is known that genetic factors and lifestyle are involved in the progression of Alzheimer’s disease [35]. In this study, there is no information on known risk factors, such as family history, head trauma, smoking, alcohol consumption, obesity, and physical activity. Adding this information would make it possible to estimate risk in more detail. Despite these limitations, this study’s findings are grounded in a large-scale, real-world dataset representative of Japan, and the results are reasonable.

## 5. Conclusions

In the treatment of Alzheimer’s disease, the incidence of adverse events is associated with the number of concurrent medications and the patterns of Alzheimer’s disease drug combinations. Both factors were shown to be independent risk factors. Although the development of therapeutic drugs is continuing, for the time being, these four drugs are considered to be the mainstay of drug therapy. By considering not only the characteristics of individual drugs but also the combination of drugs and the number of concomitant drugs, it may be possible to reduce the adverse events caused by drugs for Alzheimer’s disease. In the future, the digitization of medical information will make it easier to use large-scale real-world data, and by using these, it will be possible to promote even more precise research.

## Figures and Tables

**Table 1:** Major and mild neurocognitive disorders.

Alzheimer’s disease
Frontotemporal lobar degeneration
Lewy body disease
Vascular disease
Traumatic brain injury
Substance/medication use
HIV infection
Prion disease
Parkinson’s disease
Huntington’s disease
Another medical condition
Multiple etiologies
Unspecified

Source: American Psychiatric Association: DSM-5 [2].

**Table 2:** Mechanism and pharmacokinetics of four drugs used in Alzheimer’s disease treatment.

Drug	Mechanism	Pharmacokinetics
Donepezil	AChE inhibitor	Absorbed orally, reaching peak plasma concentration in about 3–5 h. Metabolized primarily by CYP2D6 and CYP3A4 in the liver. Excreted mainly in the urine. Half-life is 70–80 h.
Galantamine	AChE inhibitor APL action on nAchR	Absorbed orally, reaching peak plasma concentration in 1 h. Metabolized by CYP2D6 and CYP3A4 in the liver. Excreted mainly in the urine. Half-life is 5–7 h.
Rivastigmine	AChE inhibitor BuChE inhibitor	Administered orally or via patch. Metabolized by esterases. The half-life is about 1.5 h for oral administration and 3.4 h for patches. Excreted primarily in urine.
Memantine	Glutamate NMDA receptor antagonist	Absorbed orally, reaching peak plasma concentration in 1–7 h. Excreted primarily in urine. The half-life is 60–80 h.

Source: Japanese Society of Neurology: Clinical Practice Guideline for Dementia 2017 [4]. AChE: acetylcholinesterase, BuChE: butyrylcholinesterase, nAChRs: nicotinic acetylcholine receptors. APL: allosterically potentiating ligand. NMDA: N-methyl D-aspartate.

**Table 3:** Characteristics of the sample (total of 2653 cases).

Variables		*n*	%
Sex	Male	1055	39.8%
	Female	1598	60.2%
Age	60–69	131	4.9%
	70–79	786	29.6%
	80–89	1420	53.5%
	90+	316	11.9%
Combination with Alzheimer’s disease therapeutics	Memantine	207	7.8%
	Donepezil	1089	41.0%
	Rivastigmine	664	25.0%
	Galantamine	458	17.3%
	Memantine+AChEI	235	8.9%
The number of concomitant medications used	0 drugs	463	17.5%
	1 drug	271	10.2%
	2 drugs	227	8.6%
	3 drugs	264	10.0%
	4 drugs	162	6.1%
	5 drugs or more	1266	47.7%
Comorbidity	Diabetes mellitus	325	12.3%
	Hypertension	935	35.2%
	Hyperlipidemia	360	13.6%
	Ischemic heart diseases	162	6.1%
	Cerebrovascular diseases	283	10.7%
	Malignant neoplasms	82	3.1%
	Depression	124	4.7%
	Parkinson’s disease	113	4.3%
	Sleep disorders	197	7.4%

AChEI: Acetylcholinesterase inhibitor.

**Table 4:** Number of adverse events.

	Number of Cases	Percentage	Cumulative Percentage
Bradycardia	171	6.4%	6.4%
Pneumonia	122	4.6%	11.0%
Altered state of consciousness	96	3.6%	14.7%
Seizures	93	3.5%	18.2%
Decreased appetite	93	3.5%	21.7%
Vomiting	92	3.5%	25.1%
Loss of consciousness	91	3.4%	28.6%
Fractures	91	3.4%	32.0%
Cardiac failure	84	3.2%	35.2%
Falls	80	3.0%	38.2%

**Table 5:** Association between adverse events and related factors: bivariate and multivariate analyses.

		**Bradycardia**					**Pneumonia**				
			**Bivariate**		**Multivariate**			**Bivariate**		**Multivariate**	
	**Total**	**n**	**OR**	***p***	**AOR**	***p***	**n**	**OR**	***p***	**AOR**	***p***
Sex											
Male	1055	66	ref	0.746	ref	0.920	72	ref	<0.0001	ref	<0.0001
Female	1598	105	1.05		1.02		50	0.44		0.40	
Age											
60–69	131	7	ref	0.032	ref	0.063	3	ref	0.071	ref	0.067
70–79	786	39	0.92		0.89		28	1.58		1.48	
80–89	1420	110	1.49		1.38		70	2.21		2.22	
90 and older	316	15	0.88		0.84		21	3.04		3.00	
Combination with Alzheimer’s disease therapeutics											
Memantine	201	6	ref	0.018	ref	0.016	13	ref	<0.0001	ref	<0.0001
Donepezil	1006	83	2.76		3.07		25	0.35		0.37	
Rivastigmine	632	32	1.70		2.00		60	1.48		1.43	
Galantamine	423	35	2.77		3.12		21	0.72		0.73	
Memantine + AChEI	220	15	2.28		2.49		3	0.19		0.19	
The number of concomitant medications used											
0 drugs	163	20	ref	0.201	ref	0.207	31	ref	0.201	ref	0.845
1 drug	271	20	1.76		1.65		13	0.70		0.79	
2 drugs	227	15	1.57		1.36		8	0.51		0.82	
3 drugs	264	24	2.22		2.16		10	0.55		0.75	
4 drugs	162	9	1.30		1.03		4	0.35		0.64	
5 drugs or more	1266	83	1.55		1.38		56	0.64		1.02	
Comorbidities											
Diabetes mellitus	325	23	1.12	0.625	0.93	0.828	11	0.70	0.246	0.72	0.319
Hypertension	935	86	1.95	<0.0001	2.00	<0.0001	27	0.51	0.001	0.55	0.013
Hyperlipidemia	360	21	0.89	0.611	0.66	0.083	14	0.82	0.480	1.45	0.257
Ischemic heart diseases	162	16	1.65	0.069	1.48	0.196	6	0.79	0.563	0.90	0.803
Cerebrovascular diseases	283	15	0.79	0.408	0.67	0.141	11	0.82	0.536	0.97	0.937
Malignant neoplasms	82	2	0.36	0.170	0.31	0.056	9	2.68	0.016	2.16	0.066
Depression	124	4	0.47	0.186	0.48	0.125	6	1.06	0.897	1.55	0.356
Parkinson’s disease	113	4	0.52	0.206	0.57	0.235	11	2.36	0.019	2.29	0.031
Sleep disorders	197	9	0.68	0.268	0.67	0.235	4	0.41	0.047	0.43	0.077
		**Altered State of Consciousness**					**Seizures**				
			**Bivariate**		**Multivariate**			**Bivariate**		**Multivariate**	
	**Total**	**n**	**OR**	***p***	**AOR**	***p***	**n**	**OR**	***p***	**AOR**	***p***
Sex											
Male	1055	36	ref	0.643	ref	0.612	42	ref	0.282	ref	0.234
Female	1598	60	1.10		1.12		51	0.80		0.77	
Age											
60–69	131	7	ref	0.386	ref	0.419	6	ref	0.881	ref	0.793
70–79	786	25	0.58		0.58		29	0.80		0.78	
80–89	1420	56	0.73		0.71		48	0.73		0.68	
90 and older	316	8	0.46		0.45		10	0.68		0.62	
Combination with Alzheimer’s disease therapeutics											
Memantine	201	13	ref	0.096	ref	0.502	11	ref	0.058	ref	0.103
Donepezil	1006	43	0.61		0.74		45	0.77		0.84	
Rivastigmine	632	15	0.34		0.51		15	0.41		0.46	
Galantamine	423	17	0.58		0.75		11	0.44		0.47	
Memantine + AChEI	220	8	0.53		0.57		11	0.88		0.96	
The number of concomitant medications used											
0 drugs	163	2	ref	<0.0001	ref	<0.0001	14	ref	0.856	ref	0.839
1 drug	271	8	7.01		6.93		10	1.23		1.20	
2 drugs	227	6	6.26		5.90		7	1.02		0.85	
3 drugs	264	19	17.88		17.53		8	1.00		0.90	
4 drugs	162	5	7.34		6.80		4	0.81		0.63	
5 drugs or more	1266	56	10.67		10.45		50	1.32		1.13	
Comorbidities											
Diabetes mellitus	325	9	0.73	0.364	0.61	0.153	5	0.40	0.023	0.34	0.008
Hypertension	935	37	1.16	0.494	0.96	0.874	35	1.11	0.625	1.13	0.621
Hyperlipidemia	360	15	1.19	0.557	1.02	0.944	11	0.85	0.611	0.79	0.482
Ischemic heart diseases	162	7	1.22	0.631	1.09	0.829	7	1.26	0.573	1.26	0.589
Cerebrovascular diseases	283	11	1.09	0.800	0.94	0.861	12	1.25	0.489	1.23	0.533
Malignant neoplasms	82	3	1.13	1.000	0.88	0.835	1	0.33	0.367	0.32	0.175
Depression	124	5	1.53	0.803	0.93	0.881	2	0.44	0.321	0.38	0.123
Parkinson’s disease	113	6	1.53	0.300	1.43	0.437	2	0.48	0.435	0.44	0.208
Sleep disorders	197	7	0.98	0.959	0.78	0.537	11	1.71	0.126	1.68	0.149
		**Decreased Appetite**					**Vomiting**				
			**Bivariate**		**Multivariate**			**Bivariate**		**Multivariate**	
	**Total**	**n**	**OR**	***p***	**AOR**	***p***	**n**	**OR**	***p***	**AOR**	***p***
Sex											
Male	1055	35	ref	0.668	ref	0.647	18	ref	<0.0001	ref	<0.0001
Female	1598	58	1.10		1.11		74	2.80		2.71	
Age											
60–69	131	1	ref	0.351	ref	0.244	3	ref	0.680	ref	0.216
70–79	786	30	5.16		5.00		31	1.75		1.71	
80–89	1420	51	4.84		4.25		49	1.52		1.22	
90 and older	316	11	4.69		4.32		9	1.25		0.80	
Combination with Alzheimer’s disease therapeutics											
Memantine	201	5	ref	0.270	ref	0.126	5	ref	0.001	ref	<0.0001
Donepezil	1006	48	1.86		2.22		25	0.95		1.01	
Rivastigmine	632	22	1.38		2.06		39	2.52		3.56	
Galantamine	423	12	1.09		1.41		19	1.75		2.10	
Memantine + AChEI	220	6	1.06		0.99		4	0.70		0.63	
The number of concomitant medications used											
0 drugs	163	3	ref	0.000	ref	0.000	6	ref	0.011	ref	0.000
1 drug	271	9	5.27		5.06		9	2.62		3.11	
2 drugs	227	6	4.16		4.31		15	5.39		7.87	
3 drugs	264	6	3.57		3.66		9	2.69		3.78	
4 drugs	162	9	9.02		9.72		8	3.96		6.85	
5 drugs or more	1266	60	7.63		7.92		45	2.81		4.74	
Comorbidities											
Diabetes mellitus	325	14	1.28	0.415	0.99	0.970	10	0.87	0.676	0.89	0.725
Hypertension	935	44	1.68	0.015	1.18	0.468	36	1.19	0.430	1.09	0.714
Hyperlipidemia	360	17	1.45	0.195	1.06	0.831	13	1.05	0.874	0.89	0.724
Ischemic heart diseases	162	11	2.14	0.035	1.59	0.198	6	1.08	0.867	1.16	0.741
Cerebrovascular diseases	283	12	1.25	0.489	0.90	0.752	7	0.68	0.311	0.59	0.173
Malignant neoplasms	82	4	1.43	0.532	1.32	0.616	1	0.34	0.367	0.37	0.255
Depression	124	3	0.67	0.800	0.48	0.183	0	-	-	-	-
Parkinson’s disease	113	4	1.01	1.000	0.84	0.729	2	0.49	0.434	0.38	0.126
Sleep disorders	197	9	1.35	0.418	1.05	0.890	7	1.03	0.946	0.93	0.865
		**Loss of Consciousness**					**Fractures**				
			**Bivariate**		**Multivariate**			**Bivariate**		**Multivariate**	
	**Total**	**n**	**OR**	***p***	**AOR**	***p***	**n**	**OR**	***p***	**AOR**	***p***
Sex											
Male	1055	34	ref	0.632	ref	0.613	18	ref	<0.0001	ref	0.000
Female	1598	57	1.11		1.11		73	2.76		2.60	
Age											
60–69	131	7	ref	0.230	ref	0.171	1	ref	0.057	ref	0.523
70–79	786	24	0.56		0.55		22	3.74		3.24	
80–89	1420	44	0.57		0.44		52	4.94		3.47	
90 and older	316	16	0.94		0.75		16	6.93		3.47	
Combination with Alzheimer’s disease therapeutics											
Memantine	201	16	ref	<0.0001	ref	<0.0001	12	ref	<0.0001	ref	0.000
Donepezil	1006	13	0.14		0.15		16	0.24		0.28	
Rivastigmine	632	16	0.29		0.34		36	0.93		0.99	
Galantamine	423	31	0.87		0.96		19	0.70		0.80	
Memantine + AChEI	220	15	0.81		0.83		8	0.57		0.62	
The number of concomitant medications used											
0 drugs	163	7	ref	0.126	ref	0.318	18	ref	0.143	ref	0.184
1 drug	271	8	1.98		2.27		11	1.05		1.21	
2 drugs	227	10	3.00		3.18		3	0.33		0.42	
3 drugs	264	9	2.30		2.18		5	0.48		0.59	
4 drugs	162	6	2.51		2.28		4	0.63		0.87	
5 drugs or more	1266	51	2.73		2.25		50	1.02		1.31	
Comorbidities											
Diabetes mellitus	325	8	0.68	0.285	0.65	0.244	5	0.41	0.027	0.50	0.108
Hypertension	935	36	1.21	0.384	1.00	0.998	35	1.15	0.516	1.25	0.363
Hyperlipidemia	360	14	1.16	0.613	0.97	0.928	7	0.52	0.074	0.45	0.033
Ischemic heart diseases	162	13	2.70	0.004	3.01	0.003	5	0.89	0.801	1.02	0.975
Cerebrovascular diseases	283	16	1.83	0.044	1.87	0.050	7	0.69	0.328	0.71	0.386
Malignant neoplasms	82	2	0.70	1.000	0.74	0.678	1	0.34	0.528	0.37	0.258
Depression	124	2	0.45	0.442	0.51	0.308	1	0.22	0.127	0.22	0.059
Parkinson’s disease	113	3	0.76	1.000	0.82	0.733	4	1.03	0.794	1.05	0.931
Sleep disorders	197	4	0.56	0.228	0.47	0.118	10	1.57	0.191	1.57	0.240
		**Cardiac Failure**					**Falls**				
			**Bivariate**		**Multivariate**			**Bivariate**		**Multivariate**	
	**Total**	**n**	**OR**	***p***	**AOR**	***p***	**n**	**OR**	***p***	**AOR**	***p***
Sex											
Male	1055	32	ref	0.751	ref	0.850	24	ref	0.065	ref	0.129
Female	1598	52	1.08		1.05		56	1.56		1.47	
Age											
60–69	131	2	ref	0.007	ref	0.006	3	ref	0.587	ref	0.749
70–79	786	23	1.94		2.00		19	1.06		0.88	
80–89	1420	38	1.77		1.78		48	1.49		1.02	
90 and older	316	21	4.59		4.80		10	1.39		0.70	
Combination with Alzheimer’s disease therapeutics											
Memantine	201	4	ref	0.708	ref	0.607	12	ref	<0.0001	ref	0.000
Donepezil	1006	36	1.74		2.04		14	0.21		0.28	
Rivastigmine	632	20	1.58		1.64		25	0.64		0.98	
Galantamine	423	14	1.60		1.80		15	0.55		0.76	
Memantine + AChEI	220	10	2.26		2.27		14	1.03		1.16	
The number of concomitant medications used											
0 drug	163	14	ref	0.670	ref	0.594	4	ref	0.004	ref	0.002
1 drug	271	7	0.85		0.82		10	4.40		5.33	
2 drugs	227	6	0.87		0.92		5	2.58		3.04	
3 drugs	264	5	0.62		0.61		8	3.59		4.49	
4 drugs	162	6	1.23		1.37		2	1.43		1.72	
5 drugs or more	1266	46	1.21		1.28		51	4.82		5.95	
Comorbidities											
Diabetes mellitus	325	13	1.32	0.375	1.41	0.301	4	0.37	0.025	0.35	0.022
Hypertension	935	29	0.97	0.888	0.84	0.486	30	1.11	0.669	1.00	0.986
Hyperlipidemia	360	10	0.86	0.645	0.79	0.511	9	0.80	0.528	0.70	0.322
Ischemic heart diseases	162	6	1.19	0.694	1.06	0.893	2	0.39	0.234	0.39	0.142
Cerebrovascular diseases	283	10	1.14	0.713	1.15	0.698	7	0.80	0.562	0.76	0.502
Malignant neoplasms	82	4	1.60	0.329	1.62	0.396	1	0.39	0.516	0.37	0.259
Depression	124	3	0.75	0.797	0.70	0.540	1	0.25	0.181	0.22	0.056
Parkinson’s disease	113	1	0.26	0.264	0.28	0.117	1	0.28	0.259	0.24	0.077
Sleep disorders	197	7	1.14	0.751	0.97	0.939	12	2.28	0.002	2.15	0.035

The analysis was performed using logistic regression, followed by a likelihood ratio test. In bivariate analysis, Fisher’s exact test was applied when more than 20% of the cells had an expected frequency of 5 or fewer. For comorbidities, the absence of disease was used as the reference. ref: reference, OR: odds ratio, AOR; adjusted odds ratio.

## Data Availability

The data used in this study were obtained from the publicly available JADER database, which is managed by the Pharmaceuticals and Medical Devices Agency (PMDA). The dataset can be accessed through the following link: http://www.info.pmda.go.jp/fukusayoudb/CsvDownload.jsp (accessed on 21 August 2024) (only in Japanese).
